# A new device for monitoring individual activity rhythms of honey bees reveals critical effects of the social environment on behavior

**DOI:** 10.1007/s00359-016-1103-2

**Published:** 2016-07-05

**Authors:** Katharina Beer, Ingolf Steffan-Dewenter, Stephan Härtel, Charlotte Helfrich-Förster

**Affiliations:** Neurobiology and Genetics, Theodor-Boveri Institute, Biocenter, University of Würzburg, Am Hubland, 97074 Würzburg, Germany; Department of Animal Ecology and Tropical Biology, Theodor-Boveri Institute, Biocenter, University of Würzburg, Am Hubland, 97074 Würzburg, Germany

**Keywords:** Social entrainment, Foragers, Nurses, Locomotor activity, Temperature rhythms

## Abstract

Chronobiological studies of individual activity rhythms in social insects can be constrained by the artificial isolation of individuals from their social context. We present a new experimental set-up that simultaneously measures the temperature rhythm in a queen-less but brood raising mini colony and the walking activity rhythms of singly kept honey bees that have indirect social contact with it. Our approach enables monitoring of individual bees in the social context of a mini colony under controlled laboratory conditions. In a pilot experiment, we show that social contact with the mini colony improves the survival of monitored young individuals and affects locomotor activity patterns of young and old bees. When exposed to conflicting Zeitgebers consisting of a light–dark (LD) cycle that is phase-delayed with respect to the mini colony rhythm, rhythms of young and old bees are socially synchronized with the mini colony rhythm, whereas isolated bees synchronize to the LD cycle. We conclude that the social environment is a stronger Zeitgeber than the LD cycle and that our new experimental set-up is well suited for studying the mechanisms of social entrainment in honey bees.

## Introduction

Like all animals, the honey bee (*Apis mellifera*) has to cope with the daily changes in its environment and has therefore developed an endogenous clock with a period of approximately 24 h. Adapted to diurnal floral resource availability, honey bees forage throughout the day whereby foraging activity can be entrained by light-dark (LD) cycles and persists under constant conditions in the laboratory meaning that it is of circadian nature (Frisch and Koeniger 1994; Ludin et al. 2012). Honey bees also use their circadian clock for time compensated sun compass orientation (Beier and Lindauer 1970) while consulting an integrated, metric cognitive map during their homing flights (Cheeseman et al. 2014). Like all circadian clocks, the bee clock responds to many environmental cues such as light, temperature and humidity (Fuchikawa and Shimizu 2007a; Ludin et al. 2012) and can be reset by anesthesia (Medugorac and Lindauer 1967; Cheeseman et al. 2012). A special phenomenon of the bee clock is its ability of social synchronization that was elegantly demonstrated by Frisch and Koeniger (1994) but also shown by earlier studies (Medugorac and Lindauer 1967; Moritz and Sakofski 1991). Social synchronization is very important for eusocial living insect communities in which the individuals have different tasks. The bee community requires synchronization of activities on different levels: worker bees synchronize their different tasks in the beehive, e.g., receiving nectar collected by the foraging bees as well as donating food to foraging nest mates (Crailsheim et al. 1996, 1999), and conduct “social foraging” by concertedly allocating foragers to floral resources (Seeley 1986) whereas queen bees and the males (drones) go on mating flights at defined times of the day (Koeniger et al. 1996).

Analogously to the circadian rhythms of single bees, the collective rhythmic activity of bees in a colony can be shifted by light, temperature, feeding cycles and social Zeitgebers (Kefuss and Nye 1970; Frisch and Aschoff 1987; Moore and Rankin 1993; Moritz and Kryger 1994; Frisch and Koeniger 1994). It was discovered that both worker and queen bees can function as social Zeitgebers (Southwick and Moritz 1987; Moritz and Sakofski 1991) and shift the activity rhythm in the hive. In spite of the colony’s rhythmic activity, there are also arrhythmic individuals in the hive: nurse bees do not exhibit daily activity but are active and rest at rather random intervals (Moore et al. 1998), which is probably due to the fact that attending the larvae is an around-the-clock business. The transition from nursing as one of the first tasks in a honey bee’s life to foraging goes along with a change in rhythmicity, whereby the onset of rhythmicity differs between individuals and populations and can range from a few days to 3 weeks, depending on the social context and fulfilled tasks (Moore et al. 1998; Bloch et al. 2001; Eban-Rothschild et al. 2012).

Recently Giannoni-Guzman et al. (2014) have presented a locomotor activity monitoring set-up for honey bees, focusing on individually isolated animals, not taking account of the social environment, which is crucial for eusocial living insects. Here we present a new method for monitoring individual activity rhythms of honey bees in the social context of their colony. We place individually monitored bees in indirect social contact with a mini colony and therefore establish an artificial social environment under controlled laboratory conditions. We demonstrate in a pilot study with a survival assay and an experiment with conflicting Zeitgebers the importance of considering the social context when monitoring circadian activity of eusocial insects like honey bees.

## Material and methods

### Animals

Honey bee colonies are kept at the University of Würzburg, Department of Animal Ecology and Tropical Biology. They are specified as *Apis mellifera*, subspecies *carnica*. Queen mating occurred naturally.

### Experimental procedure and recording set-up

**Fig. 1:**
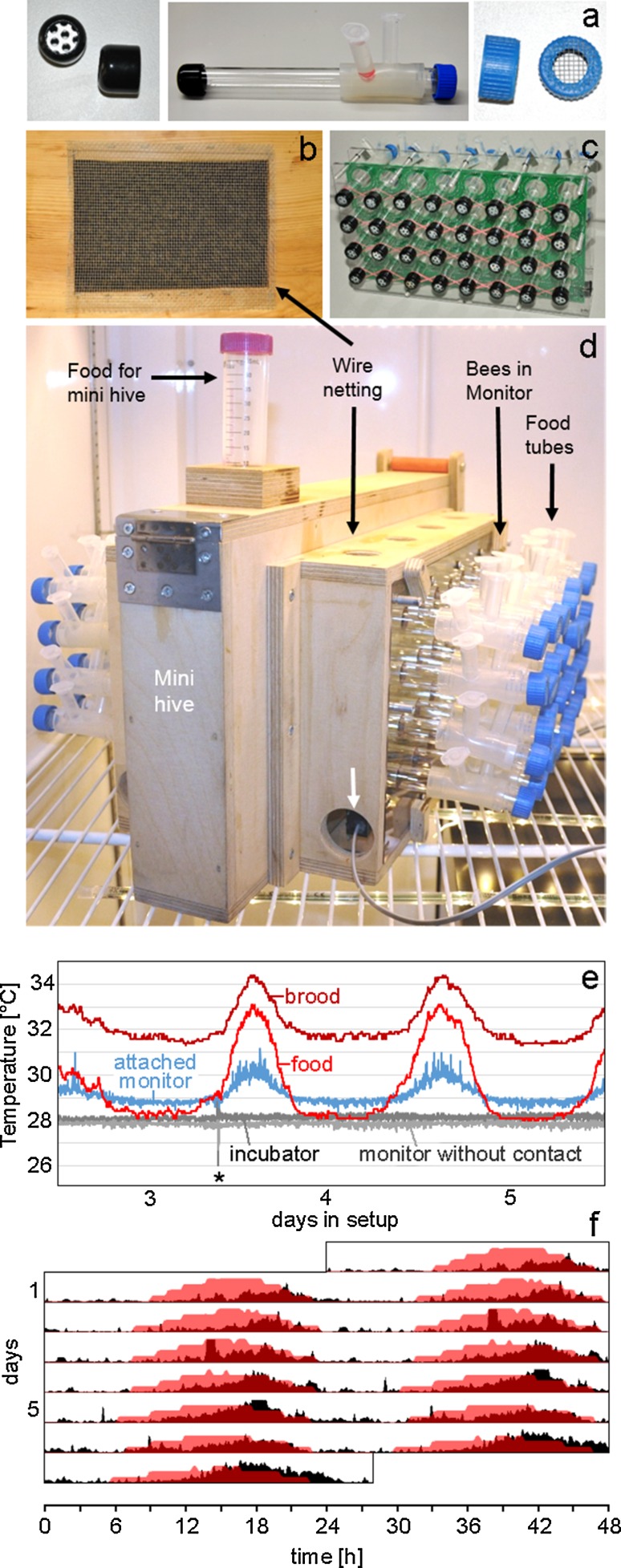
Photographs of the activity monitoring set-up in social context and examples of measured data. **a** Glass tube with ventilation caps on both endings and individual food supply for monitoring bee activity. **b** Wooden mini hive box with wire netting on the sides containing the brood comb. **c** 32 glass tubes with bees placed in the locomotor activity monitor. **d** Complete set-up. The monitors are connected to a computer interface (*white arrow*). **e** Temperature data of sensors located at different places in the experimental set-up. The *asterisk* indicates that the incubator was opened to refill the food tube of the mini hive. **f** Actogram (*double plot*) showing the circadian temperature oscillation in the hive (measured at the food sensor, *red*) together with the average activity of forager-aged bees (*black*) (*n* = 7) that are in social contact to the mini colony under DD conditions

The locomotor walking activity measurements are conducted with a system by TriKinetics (TriKinetics Inc Waltham, MA USA) based on infrared light (IR) beam barriers, which we adapted to our social context experiments (Fig. 1).

To control for age, newly emerged honey bees are marked in the course of 6 h and immediately put back into the outdoor hive. 13 days later, around the time of foraging onset, the subjects (age 13 days) are taken into the laboratory and put into monitors (see set-up photos Fig. 1d). Newly emerged bees (<6 h old) are also placed into monitors. In the experiments, these groups are referred to as forager-aged bees and nurse-aged bees. At sunrise, a brood comb (open and closed brood) with hive bees and forager bees on it (700–1000 bees estimated according to Gerig (1983)), is taken into the laboratory in a wooden box with a wire netting on the sides (Fig. 1b). Queen pheromone (Bee Boost with queen mandibular pheromone) substitutes a real queen in this mini hive to reduce stress caused by removing the bees from the hive and their queen. Bees in the monitors (adapted TriKinetics LAM16 (Locomotor Activity Monitors)) are placed next to the wire netting of the mini hive and are separated from it by a double mesh system consisting of a ventilation cap at the end of the monitoring tube (Fig. 1a) and the wire netting (Fig. 1b), which allows them to have contact with the mini colony over pheromones and microclimate (e.g., temperature, humidity, CO_2_) as well as vision and vibration, but no direct tactile contact. Control subjects socially isolated from the mini colony are placed in the same incubator, 0.5 m away from the mini hive.

To monitor the rhythm of the mini colony, temperature sensors are placed close to the brood cells, close to the food storing cells and into a tube in the monitor next to the mini hive. As controls, further sensors are placed into a tube in the monitor 0.5 m away from the mini hive and separately in the incubator. Due to space limitation in the incubator, we decided against monitoring the foraging rhythm of the colony via IR barrier beams at an entrance to a foraging arena as described in other studies (e.g., Cheeseman et al. 2012). Furthermore an IR barrier beam inside the box would be error prone due to possible blocking of the beam by a dead animal. As levels of temperature and activity both in the hive and the individual bees correlate (Kronenberg and Heller 1982; Fuchikawa and Shimizu 2007a), monitoring the temperature of the colony is a way of indirectly measuring the colony activity. A great advantage of measuring temperature is, that by allocating sensors in different places of the hive one can easily monitor different thermoregulatory activity patterns, not only foraging activity.

The food supply for individually monitored bees consists of 2 different diets, both ad libitum: a) sugar syrup by Apiinvert (Südzucker AG, 62028 Mannheim, Germany) and b) a mixture of pestled pollen pellets and Apiinvert sugar syrup (1:3). This way, the animals can choose the amount of pollen diet suitable for their age. The mini hive has pollen and honey stored in the comb cells around the brood and an extra food supply of Apiinvert sugar syrup, which serves as foraging resource and is refilled every 3rd–4th day in irregular intervals in order to prevent entrainment by feeding cycles. While refilling the additional food tube for the hive, a 3 °C temperature drop occurs, but the temperature is restored to prior levels in less than 5 min (Fig. 1e). Temperature and humidity conditions in the incubator (Percival INTELLUS, CLF Plant Climatics GmbH, 86637 Wertingen, Germany) are otherwise constant (50 % RH (relative humidity); 30 °C in the DD survival experiment, 28 °C in the LD survival study and the experiment with conflicting Zeitgebers (Fig. 1e). The illumination of monitoring tubes during the artificial light phase is between 380 and 500 lux, depending on the position of the tube; light intensity inside the mini hive box directly at the double mesh to the monitoring tubes is 7–8 lux.

### Survival tests

To determine survival rates of the bees, we place the animals in activity monitors either with or without social contact and monitor their activity until it ceases. The last signal created by a bee crossing the IR beam barrier is taken as approximate time of death, because in previous experiments we have observed that bees died in the course of a few hours after the last crossing.

### Data analysis

Activity data analysis is carried out with ActogramJ [© Benjamin Schmid and Taishi Yoshii, University of Würzburg, Department of Neurobiology and Genetics; Fiji ImageJ (Version 1.49, © Wayne Rasband, National Institutes of Health, USA)] with a data bin size in activity detection of 1 min. We determine the power of activity above significance level for the prevalent period during the first 7 days in LD with a periodogram analysis (Chi-square method, *p* level 0.05, Gaussian smoothing factor 10). The acrophase (center of gravity in activity) is calculated with the acrophase tool. We use the Wilcoxon signed rank test to compare the free-running period (τ) of individual bees and of the mini colony during DD phase, as well as to compare individual acrophases with the time point of the peak in the hive temperature. In case of “nurse-aged bees in social contact” we determine 2 activity peaks by eye after smoothing the activity signals (factor 10) of day 7 in LD, because the overall acrophase determination is inaccurate in this case. The numbers of days required by forager-aged bees to shift their activity phase to the new environmental conditions in the incubator and the days when nurse-aged bees start to show activity rhythms are identified by inspecting the individual actograms. Statistical analysis is conducted in Microsoft Excel (2013 Microsoft Office) and R (version R i386 3.2.2). Temperature and humidity data are collected with sensors from MSR (MSR Electronics GmbH, 8472 Seuzach, Switzerland; software version MSR 5.24.02) and LabJack (LabJack Corporation, Lakewood, CO 80227 USA; software version LJLog UD V1.18).

## Results

### The set-up allows simultaneous monitoring of the mini colony rhythm and of individual bees

The aim of our new approach (Fig. 1) is to include a colony environment for individually monitored bees and to simultaneously measure the rhythm of the mini colony and of individual bees. First, we had to ensure that the mini colony functioned in a comparable way to a usual colony throughout the duration of the experiments. To do so, we kept the mini hive in an incubator for 3 weeks and then checked the status of the brood. After this time, we opened the mini hive and inspected the bees and the brood cells. We found that the brood was raised normally, no dead brood was lying in the box, brood cells were opened and young bees were present in the mini colony. Only old bees that had been foragers at the start of the experiment had died, which may coincide with their natural time of death.

### Observing circadian rhythmicity within the colony

We observe clear temperature rhythms in the mini hive, with a temperature maximum in the afternoon under entrained conditions and a free-running period of 23.9 h under constant darkness (DD) (see Fig. 1f). The temperature rhythm slowly starts dampening after 2 weeks of recording in DD. Therefore, we performed our experiments during the first 2 weeks, when the temperature rhythm in the mini hive is robust.

The highest amplitude of the temperature rhythm (28–33.4 °C) is registered inside the mini hive close to the food storing cells (sensor “food”, Fig. 1e). Directly in the center of the brood the amplitude is lower, but overall the temperature is higher (31.1–34.8 °C). Temperature oscillations in the tubes of the monitor next to the brood of the mini hive have the same amplitude as at the brood center, but the average temperature is about 4 °C lower, probably because the hive temperature has to carry over a distance of c.a. 3 cm to the sensors in the glass tube (Fig. 1e). No temperature oscillations occur in the incubator and in the glass tubes of bees that are kept at a 0.5 m distance from the mini hive (Fig. 1e). Here, the temperature stays constant at around 28 °C, matching the incubator settings.

When monitoring the activity rhythms of individual honey bees with indirect social contact to the mini colony in parallel to the colony temperature rhythm, we find that the two rhythms free-run highly synchronously with a period τ of 23.9 (±0.4) h, which coincides with free-running periods in another study (Ludin et al. 2012) (no significant differences between τ of the colony and the individual bees: *p* > 0.5). Here the maximum of locomotor activity of the individual bees slightly phase-lags the maximum of the colony temperature rhythm (Fig. 1f). This indicates a synchronization of the bee activity patterns via indirectly operating social cues connected with the locomotor activity of the colony, e.g., temperature.

### The set-up improves initial survival of newly emerged bees

**Fig. 2:**
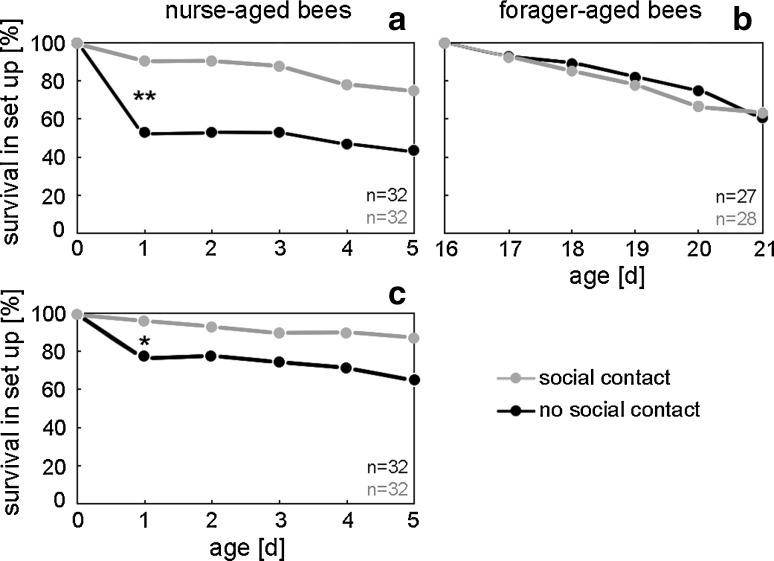
Survival of individually monitored honey bees. Newly emerged bees (nurse-aged bees) show a significant difference in survival on the first day when placed individually in glass tubes that have no contact to the mini colony. If they are in social contact to the mini colony they display a similar survival rate to the one of forager-aged bees, when put into the set-up. **a**, **b** Monitored in constant darkness (30 °C); **c** monitored in a 14:10 LD cycle (28 °C)

In order to test whether our new set-up improves bee survival, we placed two monitors, one with “forager-aged bees” and one with “nurse-aged bees” (newly emerged), next to the mini hive and two monitors of the same composition 0.5 m away from it and measured the lifespan of individuals (sample sizes: “forager-aged bees with social contact” = 28, “forager-aged bees with no social contact” = 27, “nurse-aged bees with social contact” = 32, “nurse-aged bees with no social contact” = 32). We find that significantly fewer newly emerged bees die in the first day of isolation when placed in social contact with other bees in the mini hive (Fig. 2) (Fisher’s exact test, *p* = 0.001). Their survival during the first day increases to more than 90 % and thus, is indistinguishable from the survival of forager-aged bees in the set-up (Fig. 2). From day 2 onwards survival analysis does not detect a positive effect of social contact on test bee survival. The experiments shown in Fig. 2a and b are conducted in DD, but we observe the same trend under LD cycles (Fig. 2c) (*p* < 0.05) even under slightly different incubator settings.

### Social cues are stronger Zeitgebers than LD cycles

**Fig. 3:**
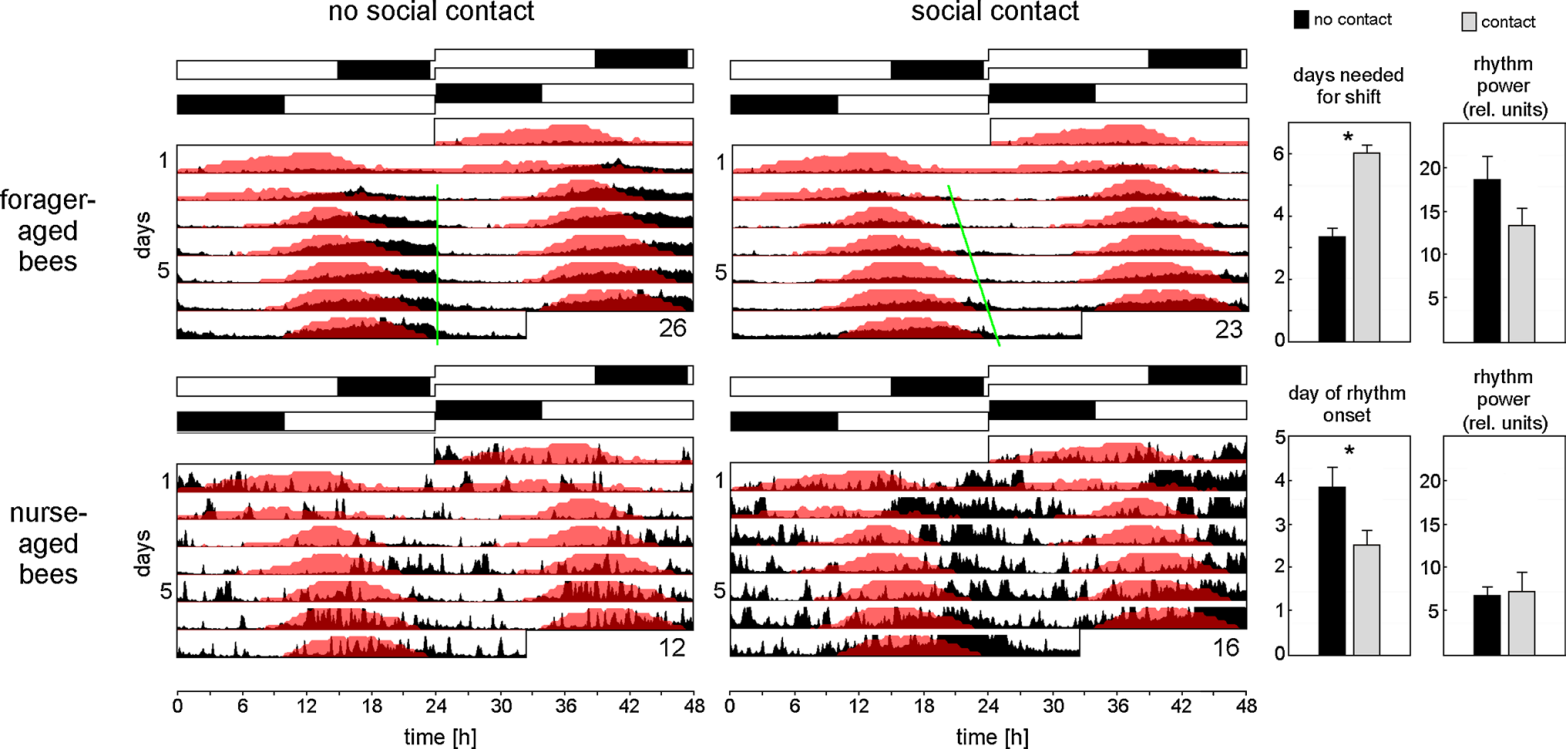
Average actograms of individually monitored bees and temperature rhythms in the mini hive after a phase delay of the LD cycle. The actograms (*double plots*) show the circadian temperature oscillations in the hive in *red* together with the average activity of the four different bee groups in *black* (forager-aged bees and nurse-aged bees without and with social contact, respectively; sample sizes are indicated in the *right bottom* of the actograms). The naturally occurring LD cycle (*upper black* and *white bars*) is phase-delayed by 10 h in the incubator (*lower black* and *white bars*). Days needed for activity shift: *green line* in actograms and diagram. Other diagrams: onset of rhythmic activity in nurse-aged bees and power of rhythmicity in forager- and nurse-aged bees

**Fig. 4:**
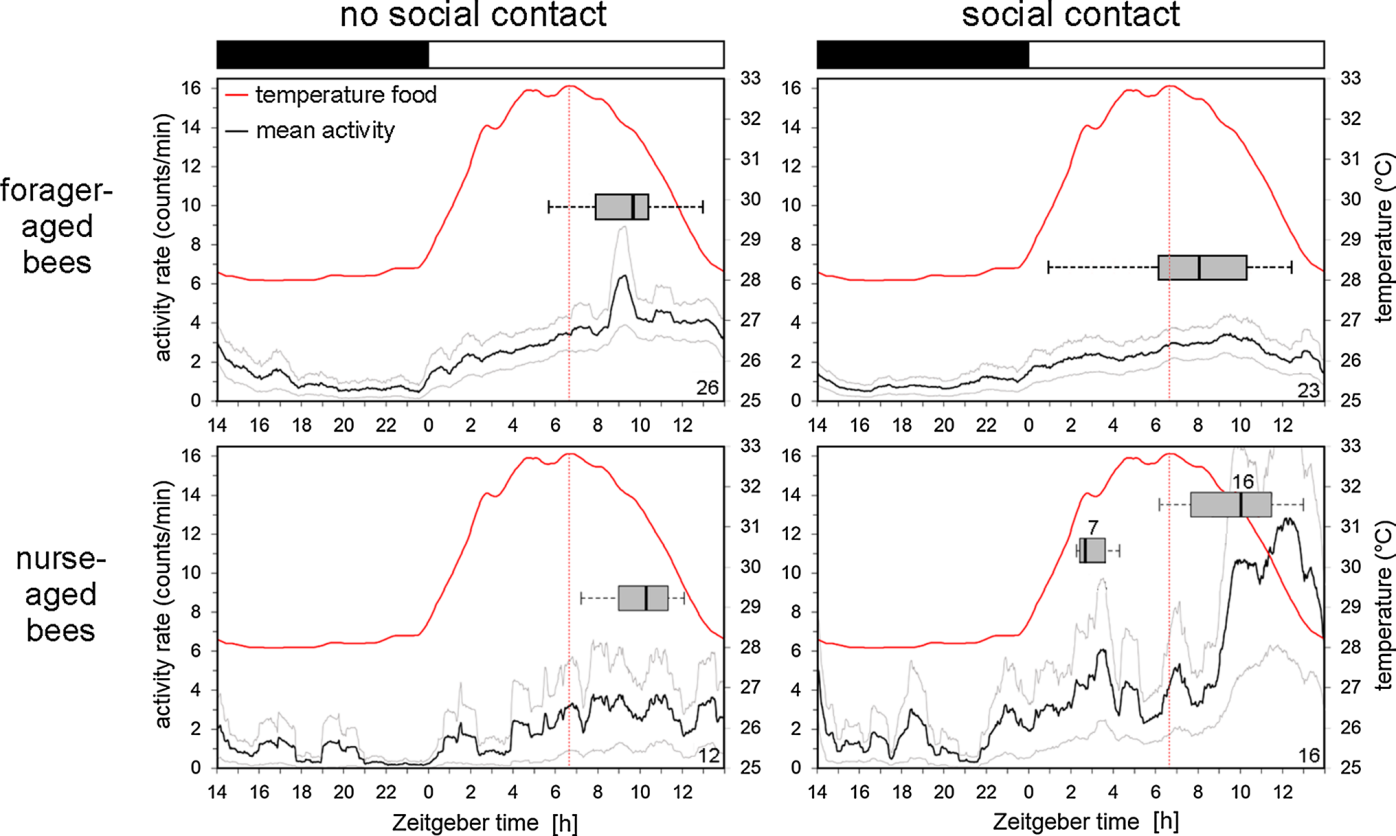
Mean activity profiles of monitored bees and temperature rhythm profile of the mini colony on day 7 in the set-up. The mean activity rate is shown as a *black line* (*gray line* = SD) of forager- and nurse-aged bees without and with social contact with the mini colony. The temperature curve of the sensor “Food” inside the mini hive is plotted in  °C as *red line*. It peaks at ZT 6.65 (*dotted red line*). Acrophases of monitored bees on day 7 in the set-up are represented as *boxplots* (sample sizes are indicated in the *right bottom* of the diagrams). The *white/black bars* on *top* of the diagrams represent the LD cycle

To test the influence of the mini colony on the activity pattern of nurse-aged and forager-aged bees, we perform an experiment in which we expose the subjects to conflicting Zeitgebers. Individual foragers, newly emerged bees and the colony for the mini hive are taken from an outdoor hive (sunrise: 05:37 o’clock; sunset: 20:56 o’clock) and placed in an incubator in which the LD cycle is delayed by approximately 10 h compared to natural conditions (Fig. 3). In the wooden box of the mini hive, the light intensity of the LD cycle is so low (≤7–8 lux), that it causes the bees in the hive to delay their temperature rhythm only slowly (Fig. 3): they need almost 7 days to adapt to the new light schedule. On the other hand, individual foragers and newly emerged bees are exposed to a LD cycle of 380–500 lux that serves as strong Zeitgeber. We thus create a situation in which bees in social contact with the mini colony have to choose between following the new LD cycle or the rhythm of the mini colony. The animals in the monitors that are kept separately from the mini hive serve as control groups. When we compare average actograms (Fig. 3) of bees with “no social contact” with the mini colony and bees with “social contact”, we see that the forager-aged bees in both groups shift their main activity from the natural light phase (see day 1) to the artificial light phase (see day 7) in the incubator. However, there is a significant difference in the speed at which they do so (*p* < 0.001, Wilcoxon rank sum test). Forager-aged bees without social contact to the colony need, on average, 3 (±0.3) days to phase delay their activity rhythm, whereas forager-aged bees in social contact with the colony mostly shift their activity rhythms in parallel with the mini colony and take on average 6 (±0.2) days to reach the new phase (Fig. 3). To compare the activity pattern of the two bee groups in a quantitative manner, we calculate the acrophase of individual bees on day 7. In forager-aged bees in social contact with the mini colony the average acrophase slightly lags the maximum of the colony temperature rhythm (Fig. 4) as already found for free-running bees in social contact (see Fig. 1), but the phases of the activity and temperature rhythms are not significantly different from each other, i.e., they are in synchrony [ZT (forager-aged bees with social contact) = 7.91 ± 0.65, *p* > 0.5]. In contrast, the activity rhythms of forager-aged bees without social contact with the colony have an acrophase significantly later than the temperature maximum of the mini colony (Fig. 4), showing that they are not synchronized with each other [ZT (forager-aged bees no social contact): 8.99 (±0.41), *p* < 0.001; ZT (nurse-aged bees no social contact): 10.12 (±0.44), *p* < 0.001].

The newly emerged bees are put into the set-up right after emergence from their brood cell. At this stage, they have never experienced a light regime before and show no particular circadian pattern in their behavior for the first 2.5–4 days in the set-up. Even after having developed a rhythm, the power of rhythmicity of nurse-aged bees is significantly lower than those of forager-aged bees and, overall, fewer bees are rhythmic compared to the forager-aged bee observations (Table 1). As expected from our survival experiments only 12 of the initial 32 young bees that have no social contact survived until the end of the experiment and are included in the average actogram (Fig. 3). Nurse-aged bees without social contact need on average 4 days to develop a rhythm and show no strong response to the LD cycle, especially in comparison to forager-aged bees (Fig. 3). Nevertheless, on the 7th day in the incubator, the bees are in phase with the LD cycle and, very similar to forager-aged bees, the acrophase of their activity rhythm is significantly later than the temperature maximum in the mini hive, showing that the bees socially isolated from the colony do not follow the rhythm of the colony.

**Table 1:** Percentage of rhythmic bees and their rhythm power

	No social contact			Social contact		
	*n*	Rhythmicity (%)	Power of rhythm (rel. units)	*n*	Rhythmicity (%)	Power of rhythm (rel. units)
Forager-aged bees	26	84.62	18.63 ± 2.69	23	95.65	13.51 ± 1.88
Nurse-aged bees	12	58.33	6.93 ± 1.02	16	75.00	7.29 ± 1.16

As already observed for the forager-aged bees, activity patterns of the nurse-aged bees in social contact with the colony are strikingly different from the bees without social contact (Fig. 3). The former develop a rhythm already 2.5 (±0.4) days after the transfer to the monitors, which is significantly earlier (*T*_(17)_ = 2.31, *p* = 0.03), and are very active. However, activity levels are not significantly different between nurse-aged bees with and without social contact due to a high variability between individual bees. A closer inspection reveals that 7 of the 16 nurse-aged bees with social contact are extremely active (~5000 IR beam crosses per day). The others cross the IR beam on average ~ 2000 times/day and are, thus, similarly active as the nurse-aged bees without social contact (1924 IR beam crosses/day). *χ*^2^ analysis highlights that the number of individuals with high activity (>3000 IR beam crosses/day) is significantly larger in nurse-aged bees with social contact than in all other groups (*χ*^2^ = 7.58; *p* = 0.0059). Most interestingly, the highest activity of these bees always occurs when the temperature in the mini hive drops (Fig. 3). On day 7, two activity bouts are visible in the activity pattern of the nurse-aged bees with social contact, but not in the test group without social contact (Fig. 4): the one around the temperature drop in the hive, that is present in all 16 surviving subjects, and the one around the temperature rise in the hive detected in seven individuals (Fig. 4).

Together, these results indicate that the activity rhythm of the bees in social contact with the mini colony is strongly influenced by the colony’s rhythm: the social Zeitgeber appears stronger than the environmental LD cycle.

## Discussion

With our new approach to monitor rhythms in honey bees, we are able to put individual locomotor activity patterns in context with the social environment of a mini colony. We combine a red-light barrier locomotor activity monitoring system with temperature monitoring in a mini hive, which is based on a single frame system designed for honey bee colony keeping in the laboratory. We show that the mini colony mimics the social environment in a natural bee colony in some aspects, but at the same time compromises other aspects like free flight activity to the realization of this social set-up in a laboratory with limited space capacity. Nonetheless we can show that the established social contact improves survival of newly emerged bees on the first day and enables nurse-aged bees to display diurnal activity rhythms from the second day of their adult life onwards, which is significantly earlier than in nurse-aged bees without social contact. In a competition experiment with conflicting Zeitgebers, we demonstrate that in our set-up bees can socially synchronize their locomotor activity rhythms with the mini colony rhythm and social cues seem to be more important Zeitgebers than light, in both nurse-aged and forager-aged bees.

### The set-up facilitates high data through-put

Besides classical observations (Beier and Lindauer 1970; Kefuss and Nye 1970; Crailsheim et al. 1996) which are inexpensive but time-consuming, large data sets can be evaluated more efficiently with automated systems with, e.g., video tracking software (Sauer et al. 2003; Gilestro 2012; Eban-Rothschild and Bloch 2015). The disadvantages of this monitoring system are limited capacity of individual tracking, high purchase costs and susceptibility to errors, if videotaping conditions are suboptimal. We decided for an automated system based on movement detection by red-light barrier beams, because of its cost-efficiency and simplicity in application. The DAM system by TriKinetics has been successfully used for decades and facilitated high data throughput studies of locomotor activity to gather consolidated findings in the field of chronobiology (Schlichting and Helfrich-Förster 2015).

### The mini colony mimics a natural bee colony in a number of aspects

A natural bee colony is made up of thousands of individuals and especially includes a queen. The colony in the mini hive is a reduced version with up to 1000 bees and the queen is substituted with a queen pheromone. Therefore, the extent to which the mini colony, which does not include a queen and is therefore not fully functional, mimics a natural bee colony has to be tested. In former studies the absence of a queen had no effect on thermoregulation in a beehive (Kronenberg and Heller 1982). Furthermore, the following observations in our set-up are in favor of a normal colony function: (1) bees in the mini hive raise the brood normally, so that the next generation is still alive and apparently healthy after 3 weeks in the box. (2) We observe different temperature amplitudes at the brood and at the food storage cells. This resembles the situation in a natural beehive, in which honey bees typically keep their brood at constant high temperatures of approximately 32–36 °C, but put less effort in heating the food cells (Simpson 1961; Kronenberg and Heller 1982; Kleinhenz 2003; Stabentheiner et al. 2010). Forager and hive bees that have duties other than handling the brood are rhythmic and usually stay outside the brood center (Bloch et al. 2001; Stabentheiner et al. 2010), coinciding with the sites where we observe the highest temperature oscillations (at the food sensor). The bees in our set-up cannot forage outside of the mini hive box. Therefore, foragers produce daily oscillation patterns in temperature because their body temperature raises during their activity phase, as it has been observed in studies on isolated forager bees (Fuchikawa and Shimizu 2007a). The recorded brood temperature lies between 31.1 and 34.8 °C, which indicates that few nursing workers can actively regulate the brood temperature, which is important for a normal development (Tautz et al. 2003; Groh et al. 2004; Jones et al. 2005). The slight temperature drop detected by the brood sensor is probably due to the small number of bees in the mini hive. Other studies of hive temperature in single frame colonies yield similar results (Kronenberg and Heller 1982). In summary, our mini colony appears suitable to create a simulated colony environment for the singly housed bees. This is confirmed by the better initial survival of newly emerged honey bees with social contact as compared to those in isolation.

### Impact on other non-chronobiological studies in young bees

The impact of social contact on the behavior of bees is not only interesting for chronobiological studies. Newly emerged bees are very sensitive to stress and have a high mortality rate when they are caged in the laboratory (Milne 1982). In our experiments, we see a clear effect of separation stress on newly emerged individuals (day 1), which can be compensated when the newly emerged bees have indirect social contact. It has been shown that nurses promote the development of young bees probably by feeding jelly (Naiem et al. 1999). But we can show that the high mortality among the socially isolated newly emerged bees in our set-up is not related to malnutrition because all test subjects are provided with the same food and prevented from conducting trophallaxis with bees in the mini colony by a double mesh separation system. During the very early stage of adulthood separation from a social environment seems critical for survival and this is not only due to nutritional reasons. In this respect, our set-up can help to realize experiments with newly emerged bees as test subjects.

### Contact with the mini colony enables social synchronization

Most interestingly, our set-up seems to allow social synchronization of individuals with the mini colony rhythm. This is evident under DD conditions, in which the activity rhythms of forager-aged bees free-run in parallel to the temperature rhythms of the colony. To investigate whether social cues are stronger Zeitgebers than external LD cycles, we perform an experiment in which half of the subjects are exposed to conflicting social and LD cycles and the other half only to LD cycles. We find significant differences in the rhythmic behavior of these two groups, independently of their age (even though the responses of forager-aged and nurse-aged bees are quite different with respect to rhythmicity). Forager bees in isolation display exactly the same percentage of rhythmicity (84 %) as shown in an earlier study (Ludin et al. 2012), whereas the indirect social contact in our set-up raises the number of rhythmic bees in both age groups.

Forager-aged bees with social contact display an unusually low response to the light signal but instead phase delay their activity rhythm in synchrony with the hive rhythm. This clearly shows that the social Zeitgeber is stronger than the LD cycle. The overall lower activity of bees with social contact may be a response to the conflicting Zeitgebers, which give two different entraining inputs. Nurse-aged bees without social contact do not develop an immediate activity rhythm under LD cycles. They need about 7 days to synchronize with it. The small and delayed response to light may be explained by the following reasons: (1) their visual system is not fully mature, which is unlikely, because the electroretinogram of nurse-aged bees appears normal (Ben-Shahar et al. 2003). Further Ben-Shahar et al. showed that nurse-aged bees do not display a forager-like phototactic response, which speaks for reason (2) the connections between the visual system and the circadian clock are not yet established or the responsiveness to light is differently modulated in the nurse-aged bees. If so, the earlier diurnal activity patterns in nurse-aged bees with social contact may also be due to the fact that the light input cannot entrain very young bees, but social cues can. Our observation that bees need about 4 days to establish a rhythm supports a third option (3) they still lack an operating clock right after emergence, which is in line with previous studies (Bloch et al. 2001; Eban-Rothschild et al. 2012). Eban-Rothschild et al. (2012) conclude in their study that, for a proper development of the circadian clock in honey bees, exposure to the colony environment during the first 48 h after emergence is mandatory. They showed this in a comparison of two test groups: one in social contact, the other in isolation for the first 48 h of their adult life. Afterwards, isolated in the laboratory, only bees that had social contact before displayed circadian rhythms in their behavior right away. In line with this study we also observe that nurse-aged bees with social contact show diurnal activity patterns significantly earlier, namely from the second day of their adult life on. Nevertheless, we cannot exclude that the connections between the visual system and the circadian clock are additionally poorly established in young bees, or the clock network is simply not yet synchronized.

Half of the nurse-aged bees in contact with the colony start to be very active when temperature drops in the mini hive, which seems as if these bees try to counteract the drop in hive temperature. Indeed, nurses maintain temperature at the brood constant by either producing heat via clustering and indirect flight muscle contractions if the temperature is too low or by fanning if the temperature is too high (Simpson 1961; Esch and Bastian 1968; Stabentheiner et al. 2010). The bees are hindered to cluster in the monitoring tubes and therefore may attempt to produce heat via raising their locomotor activity. The fact that bees in our set-up initiate this behavior on the second day is in line with Stabentheiner et al. (2010), who showed that bees are only able to actively thermoregulate from day 2 onwards. Earlier studies show that the temperature thresholds for engaging in thermoregulation are variable in colonies with diverse genetic background, wherefore the workers are able to control hive temperature more efficiently (Jones et al. 2004; Myerscough and Oldroyd 2004). Since our colony also has a diverse genetic background by natural mating, it is likely that only some bees start heating behavior when temperatures drop below 29 °C, while others may start later.

It is surprising that both forager- and nurse-aged bees in social contact barely respond to the LD cycle, because light is a strong Zeitgeber most organisms can be entrained to (Aschoff and Pohl 1978). Moreover one could expect that especially nurses, which are used to a dark surrounding in the hive, would be particularly sensitive to light pulses. So far, only one study in honey bees indicates that social cues have more importance than light (Fuchikawa et al. 2016). In this study, a field foraging colony was exposed to conflicting LD and social cycling (foraging activity). Later put into constant conditions in the laboratory, foragers and nurses showed the same entrainment pattern to the foraging cycle, whereas a different group of caged nurse-aged bees that only experienced the LD cycles showed entrainment to the LD cycles. Fuchikawa et al. concluded that social cues override the light input in young nest bees. In addition, our study shows that social cues can be stronger Zeitgebers than light also for forager-aged honey bees as long as they are prevented from foraging outdoors.

### Putative factors responsible for social synchronization

The necessary cues for social synchronization in a beehive are yet unknown. There are indications that contact over microclimate (temperature, CO_2_, relative humidity) or vibration (Moritz and Kryger 1994; Simoni et al. 2014) as well as pheromones, which are used in nest mate communication in a variety of situations, e.g., swarming or alerting behavior (Free 1987) could play a role in the social synchronization of activity rhythms. Olfactory cues have been shown to be involved in social entrainment in rodents (Goel and Lee 1997) and, to some extent, in fruit flies (Levine et al. 2002; Krupp et al. 2008). Our bioassay can be used to unravel the exact mechanism of social entrainment in honey bees, by manipulating different putative cues for social entrainment. Due to our double mesh system between the monitored bees and the mini colony we can exclude physical contact and other studies support this conclusion (Moritz and Kryger 1994; Fuchikawa et al. 2016). Temperature cycles can be ruled out as sole synchronizing cue in our set-up as well, because we measure temperature amplitudes of ~2 °C in the glass tubes that are well below the tested minimum (6–10 °C) for complete entrainment of the circadian clock of honey bees (Moore and Rankin 1993; Fuchikawa and Shimizu 2007b).

### Outlook

Our set-up can be modified in several ways to further study social effects on the locomotor activity rhythms and entrainment of circadian rhythms of individual bees. By removing the net on the tube cap that provides the double mesh system between the tubes and the mini colony, one can monitor the effect of direct tactile contact and trophallaxis on behavior. One can prevent CO_2_ or pheromones from entering the tube by closing the side facing the mini hive with a volatile-tight membrane, or visual signals can be blocked by a shield introduced between individual bees and the colony bees. Furthermore vibrational signals can be eliminated by removing the wooden clamp that connects the monitor to the hive box and introducing a material that buffers vibrations.

Monitoring bees in indirect contact is of great advantage over monitoring in groups with tracking systems: one can easily perform manipulation studies without affecting the hive bees. This permits studies like different feeding assays in social context with the mini colony, where all bees in the monitors can have their individual food provisioning and the double mesh system prevents them from carrying out trophallaxis.

Moreover, our set-up also makes bee sampling for molecular analysis easier, as it saves the experimenter from having to open a beehive. This would be especially interesting for analysis of clock gene expression in differently aged honey bees to gain a better understanding of how the clock input works in different life stages.

In summary, we offer a new and easily applicable set-up for investigating individual locomotor activity rhythms in social context under controlled laboratory conditions. Although our set-up does not allow the bees to perform age related tasks and thus does not fully simulate the natural situation in a honey bee colony, it is well suited to study particular effects of the social environment. For example it can be used to identify the cues needed for synchronization of singly housed bees with the rhythms of the colony. Furthermore, our system allows manipulations to the bee’s social system that can be performed even in a laboratory with space limitations. Thus, our set-up could have a great impact on the realization of laboratory experiments aimed at understanding honey bee behavior and the influence of social interactions in a honey bee community.
